# Characteristics of Artificial Virus-like Particles Assembled in vitro from Potato Virus X Coat Protein and Foreign Viral RNAs

**Published:** 2011

**Authors:** M.V. Arkhipenko, E.K. Petrova, N.A. Nikitin, A.D. Protopopova, E.V. Dubrovin, I.V. Yaminskii, N.P. Rodionova, O.V. Karpova, J.G. Atabekov

**Affiliations:** Biology Department, Lomonosov Moscow State University; Advanced Technologies Center; Physical Department, Lomonosov Moscow State University; Belozersky Institute of Physico-Chemical Biology, Lomonosov Moscow State University

**Keywords:** plant viruses, RNA, viral ribonucleoproteins, translation activation

## Abstract

Potato virus X (PVX) and some other potexviruses can be reconstituted*in vitro*from viral coat protein (CP) and RNA. PVX CP is capable of forming viral ribonucleoprotein complexes (vRNP) not only with homologous, but also with foreign RNAs. This paper presents the structure and properties of vRNP assembled*in vitro*upon incubation of PVX CP and RNAs of various plant and animal viruses belonging to different taxonomic groups. We have shown that the morphology and translational properties of vRNPs containing foreign (heterologous) RNA are identical to those of homological vRNP (PVX RNA – PVX CP). Our data suggest that the assembly of the “mixed” vRNP*in vitro*could be started at the 5’-proximal region of the RNA, producing a helical structure of vRNPs with foreign nucleic acids. The formation of heterologous vRNP*in vitro*with PVX CP appears not to require a specific 5’ end RNA nucleotide sequence, and the PVX CP seems to be able to pack foreign genetic material of various sizes and compositions into artificial virus-like particles.

## INTRODUCTION

The protein capsid of a number of phytoviruses consists of identical coat protein subunits folded on the basis of helical symmetry. Genomic viral RNA is helically arranged between the turns of coat protein subunits and follows their folding. The possibility of reversible dissociation of virions into coat proteins and RNA followed by the *in vitro* self-assembly of viral ribonucleoproteins (vRNP) is an important feature of a number of viruses. As a result, the structure and biological activity of the virus can be reconstituted. The self-assembly (repolymerization) of a low-molecular-weight coat protein can occur in the absence of RNA, yielding particles with a structure identical to that of viruses, but possessing an unlimited length [3].

The self-assembly procedure enables one to obtain a “mixed” vRNP consisting of the viral coat protein and a heterologous RNA [4, 5]. The fact that it is possible to construct viruses containing foreign RNA creates the potential for using a “mixed” artificial vRNP to target cells and organs of plants and, possibly, animals with foreign RNA. It is convenient to use plant viruses to form “mixed” vRNPs, since they are highly stable, completely biologically safe (there are no pathogens common to both plants and animals) and the procedure of vRNP assembly is a low-cost one due to the exceptionally high level of accumulation of a number of viruses in an infected plant (4–10 g/kg of leaves).

Another advantage of vRNP is the possibility of controllable activation of the translation of the RNA encapsulated in the coat protein. Viruses and a “mixed” vRNP can change their structure under the effect of a number of factors (pH shift, phosphorylation, and the presence of certain virus-specific activating proteins).

The spiral viruses of plants, which are rather highly stable at high temperatures, non-physiological pH values of the environment and in the presence of hydrolytic enzymes, are preferable for the construction of vRNP. Moreover, the length of a spiral virus depends on the size of the nucleic acid; as opposed to isometric viruses, hence, vRNP formed in vitro can include RNA of unlimited length. Looking ahead, it is reasonable to assume that the spiral phytoviruses that are modified and reconstructed in a “mixed” manner can serve as containers to store and deliver “therapeutic” genes and pharmaceutical agents into cells [3].

Potato virus X (PVX) is one of the phytoviruses with a spiral structure, a typical representative of *Potexvirus* genus of the family *Flexiviridae* . PVX virions are flexible thread-like bodies 515 nm long and 13.5 nm in diameter. A viral particle contains approximately 1350 helically folded identical coat protein (CP) subunits and a viral RNA enclosed between the helix turns [6]. A turn of the primary PVX helix consists of 8.9 CP subunits. The PVX genome consists of a 6345 nt single-stranded (+)-RNA [7]. The genomic RNA contains a cap at its 5’ terminus and a poly(A) sequence at its 3’ terminus [8]. The RNA of PVX encodes five proteins: viral replicase (molecular weight of 165 kDa) and four proteins that are responsible for the intercellular and systemic transport of the infected material; three movement proteins (MP1, MP2, and MP3, the products of the “triple gene block”) with molecular weights of 25, 12, and 8 kDa, respectively; and a coat protein with a molecular weight of 25 kDa [8].

It has been shown that the coat protein of potexviruses is capable of formingvRNP *in vitro * not only with the homologous RNA, but with certain heterologous RNAs as well [9, 10].

The present study was aimed at investigating the structure and the properties of vRNP obtained *in vitro * by the incubation of PVX CP with the RNA of a number of plant and animal viruses belonging to various taxonometric groups. The RNAs of potexviruses (NMV – narcissus mosaic virus, PAMV – potato aucuba mosaic virus, AltMV – alternanthera mosaic virus), tobamovirus (TMV – tobacco mosaic virus), bromovirus (BMV – brome mosaic virus), and Mengo picornavirus (animal virus) were used as heterologous RNAs.

## EXPERIMENTAL

**Purification of PVX specimens, coat protein, MP1 and PVX RNA**

The PVX specimen (Russian strain) was extracted from infected plants * Datura stramonium L. * according to the procedure described by Atabekov *et al* . [11]. PVX CP was obtained by salt deproteinization [12]. RNA was extracted using the phenol technique [13] with modifications. Recombinant protein MP1 was obtained according to the procedure described earlier [14].

*In vitro* **obtainment of vRNP**

In order to obtain vRNP, the RNA and the coat protein were mixed at a RNA : CP weight ratio of 1 : 10. The incubation was carried out under the standard conditions [15]: in 20 µl of a 0.01 M tris-HCl buffer, pH 7.5 at room temperature for 20 min. The reaction was stopped by adding bromophenol blue or transferring the incubation mixture into ice (0°С).

*In vitro* **translation**

RNA translation was carried out in a cell-free protein-synthesis system consisting of a wheat germ extract according to the procedure described earlier [14], in the presence of ^35^ S-methionine for 60 min at 25°С. The amount of RNA in the sample was 40 µg/µl (for Mengovirus RNA, 25 µg/µl). Recombinant MP1 for the translational activation of RNA within vRNP was added at a PVX: MP1 molar ratio = 1 : 100; i.e., 1.4 µg of MP1 per 1 µg of RNA (20 µg of the virus).

**Transmission electron microscopy**

The specimens (15 µl) were sorbed onto copper grids for electron microscopy, coated with formvar film (a 0.5% formvar solution in dichloroethane was used for film coating) for 15–20 s. Then, the specimens on the grids were contrasted with a 2% uranyl acetate solution and viewed on a JEOL JEM-1011 (JEOL, Japan) electron microscope at 80 kV. The images were obtained using a Gatan Erlangshen ES500W digital camera and Gatan Digital Micrograph™ software.

**Atomic force microscopy (AFM)**

The scanning was performed on the Nanoscope 3a (Digital Instruments, Santa Barbara, United States) and SmartSPM (Aist-NT, Russia) microscopes in air in the resonance mode. The typical scan rate was 1 Hz. Cantilevers fpN01S with a resonance frequency of 118–190 kHz, rigidity of 5.3 N/m, and guaranteed needle bending radius of 10 nm (F.V. Lukin State Research Institute of Physical Problems, Russia) were used. FemtoScan Online software (Center for Advance Technologies, Russia) was used to process and visualize AFM images. For sample preparation, 5–10 µl of the specimen at the required concentration was applied to freshly cleaved mica or highly oriented pyrolytic graphite for 5–10 min. Then, the sample was washed twice in a drop of distilled water and air-dried.

## RESULTS and DISCUSSION

A series of vRNP were obtained by *in vitro * assembly of PVX CP and the RNAs of viruses belonging to various taxonomic groups. The RNAs of the following viruses were used as heterologous RNA: four potexviruses (PVX, NMV, PAMV, and AltMV), tobamovirus (TMV), bromovirus (BMV, icosahedral virus with a functionally fragmented genome), and Mengo picornavirus (animal virus). The homologous RNA of PVX was used as a control.

**Fig. 1:**
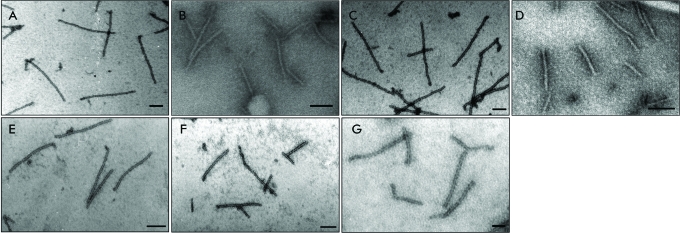
TEM images of the vRNPs assembled *in vitro* from homologous or foreign RNA and PVX CP. (A) PVX RNA;  (B) NMV RNA; (C) PAMV RNA; (D) total BMV RNA; (E) Mengo virus RNA; (F) TMV RNA; (G) AltMV RNA. The RNA : CP ratio (w/w) = 1 : 10. The samples were stained with 2% uranyl acetate. Scale bars represent 100 nm.

The PVX CP is known not to form virus-like aggregates in the absence of RNA [16]. Particles that are morphologically indistinguishable from those obtained upon the reconstruction of PVX CP with homologous PVX RNA ( *Fig. 1A* ) can be observed in a transmission electron microscope (TEM) after incubation of PVX CP with heterologous RNAs of various viruses at a RNA : CP ratio = 1 : 10 (w/w) ( *Figs. 1B–G* ). We have already demonstrated that homologous vRNP “PVX RNA – PVX CP” are structurally identical to native PVX virions [15].

**Fig. 2:**
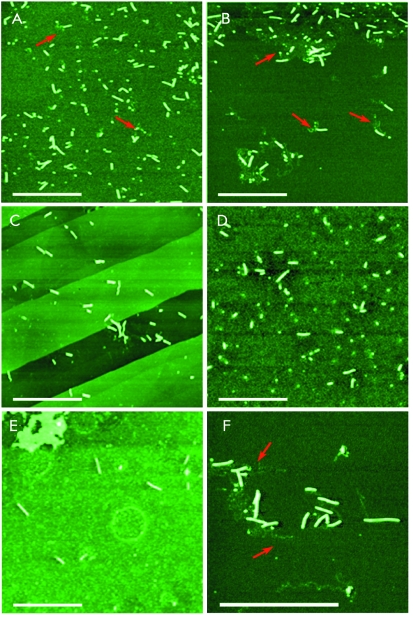
AFM images of the vRNPs assembled *in vitro* from homologous or foreign RNA and PVX CP. (A) PVX RNA on mica; (B) TMV RNA on mica; (C) NMV RNA on graphite; (D) total BMV RNA on mica; (E) Mengo virus RNA on mica; (F) PAMV RNA on mica. The RNA : CP ratio (w/w) = 1 : 10. The samples were air-dried. Cantilever oscillation frequency 300–350 kHz. Arrowheads point to protein-free RNA tails. Scale bars represent 1 μm.

**Fig. 3:**
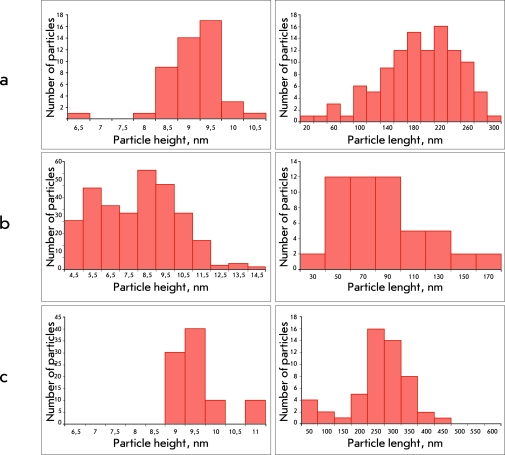
Histograms showing height and length distribution of vRNP on the basis of the AFM data. PVX CP was incubated with RNA at RNA : PVX CP (w/w) ratio = 1 : 10. (A) PVX RNA;  (B) total BMV RNA;  (C) Mengo virus RNA.

The vRNP morphology was analyzed using high-resolution AFM imaging. The vRNP particles containing homologous and heterologous RNAs were studied using this technique. The images of vRNP obtained upon the incubation of PVX CP with heterologous RNAs ( *Fig. 2B–F* ) are identical to those of homologous vRNP ( *Fig. 2A* ). The AFM data showed that the average height of a homologous and heterologous complex was 10.0 ± 0.6 and 9.9 ± 0.9 nm, respectively ( *Fig. 3* ). These values coincide with each other within the limits of error and correspond to the height of the native PVX (data not shown). As mentioned above, the diameter of the PVX virion is 13.5 nm [6]. The heights of the homologous complexes determined using the AFM technique agree with this value [17]. However, the height and width of a viral particle determined by AFM may vary depending on the type of probe used, the method of sample preparation, and the value of the force action. When performing measurements in air, the height of PVX virions is typically underestimated and equal to 10–11 nm. This is associated with the fact that during scanning, the microscope probe imposes pressure on the sample and flattens it to a certain extent [18].

TEM and AFM were used previously to detect single-tailed particles (STPs) with the 3’ terminus of PVX RNA that was vacant of CP, and rod-like heads that resulted from the helical folding of the coat protein on the 5’ terminal RNA fragment [15].

The 1 : 10 (w/w) RNA : coat protein ratio in the incubation mixture upon vRNP assembly ensures the absence of excessive unbound CP on the specimen surface. On the other hand, this amount of CP is insufficient for encapsulation of the entire RNA. As a result, AFM detects the particles in which a part of the RNA molecule within vRNP remains unbound to CP ( *Figs. 2A, B, F* ). It should be noted that not all short vRNPs have unbound RNA tails ( *Figs. 2C–E* ). This may result from the hydrolysis of the 3’ RNA terminus not bound to CP due to the action of the ribonucleases in the solution or upon loading the particle suspension on the mica surface prior to the analysis.

The self-assembly of RNA with the viral CP leads to the formation of a set of vRNPs heterogeneous in length ( *Fig. 3* ). Particles containing a completely encapsidated RNA were not found even when analyzing the homologous vRNP ( *Fig. 3A* ). The most completely reconstructed PVX vRNPs reached just 300 nm, whereas the modal length of the native virions is 515 nm. The decrease in vRNP length seems to be the result of the deficiency of the coat protein in the incubation mixture (the RNA : CP ratio was 1 : 10 instead of the 1 : 20 ratio that is used to reconstruct the full-size PVX particles).

The increase in the amount of protein within the incubation mixture (calculated per RNA molecule) results in an increase in the length of “mixed” (heterologous) particles. Thus, at a RNA : CP ratio = 1 : 10, heterologous particles formed after protein “coating” of the RNA of potexviruses NMV, PAMV, and tobamovirus TMV were characterized by an average length of 200 nm similar to that of PVX vRNP ( *Fig. 3A* ). The size of the RNA of these viruses is comparable with that of PVX RNA. When using shorter viral RNAs (the total specimen of BMV RNA consists of four RNAs with lengths varying from 800 to 3234 n), the RNA : CP molar ratio decreased while the number of short particles (80–100 nm) increased ( *Fig. 3B* ). On the other hand, upon incubation of Mengo virus RNA (8400 n) with the PVX CP, the molar ratio increased and the average size of the particles increased to 400–450 nm ( *Fig. 3B* ).

Earlier, we had revealed that the RNA molecule within native particles of PVX and homologous single-tail vRNPs (PVX RNA – PVX CP) is inaccessible for translation as opposed to TMV and a number of other viruses. However, RNA translation is activated upon phosphorylation of PVX CP or upon formation of the virion or vRNP with PVX MP1 [11, 15, 19].

In the present work, we studied the translation properties and specificity of the translation activation of “mixed” vRNPs using MP1.

**Fig. 4:**
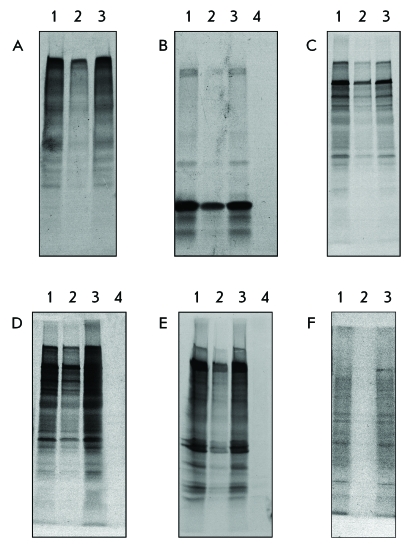
*In vitro* translational activation of RNA within vRNP. vRNP are formed upon incubation of PVX CP with homologous and foreign RNAs at weight ratio = 10 : 1, except for lane 4 at sections B, D, E, where the CP : RNA ratio = 30 : 1. Electrophoretic analysis (in 8–20% denaturating polyacrylamide gel) of ^35^ S labeled translation products produced in wheat germ extract. (A) PVX RNA; (B) total BMV RNA; (C) PAMV RNA; (D) NMV RNA; (E) TMV RNA; (F) Mengo virus RNA. Lane 1 is RNA; Lane 2 – RNA + PVX CP; Lane 3 – (RNA + PVX CP) + MP1.

It was demonstrated in the control experiments that interaction between PVX RNA and PVX CP results in inhibition of RNA translation within vRNP compared to that of unbound RNA ( *Fig. 4A, 1, 2* ). The background translation level observed ( *Fig. 4A, 2* ) may result from the presence of unbound RNA because of CP deficiency upon incubation [15]. The amount of unbound RNA decreases as the RNA : CP molar ratio increases, accompanied by a drop in the background translation level [15].

On the other hand, the interaction between MP1 and vRNP consisting of homologous coat proteins and RNA results in efficient translation activation of the encapsidated PVX RNA ( *Fig. 4A, 1, 3* ).

It is worth mentioning that similar results were obtained by analyzing the translational activation of the heterologous RNAs within the vRNPs reconstructed from PVX CP ( *Figs. 4B–F* ). The addition of PVX CP to the RNA at the 10 : 1 (w/w) ratio leads to a noticeable translation suppression of BMV RNA ( *Fig. 4B, 1, 2* ), PAMV RNA ( *Fig. 4C, 1, 2* ), NMV RNA ( *Fig. 4D, 1, 2* ), TMV RNA ( *Fig. 4E, 1, 2* ), and Mengo virus RNA *(Fig. 4F, 1, 2* ) within vRNP as compared with the same amount of unbound RNA. Almost complete translation suppression of encapsidated RNA can be achieved by increasing the amount of CP to the RNA : CP ratio = 1 : 30. Fig. 4 shows the results for BMV RNA ( *Fig. 4B, 4* ), NMV RNA ( *Fig. 4D, 4* ), and TMV RNA ( *Fig. 4E, 4* ). The addition of the MP1 to the “mixed” vRNP results in translation activation ( *Figs. 4B–F, 3* ), the translational efficiency recovering to the level of unbound RNA ( *Figs. 4B–F, 1* ). The set of peptides that are formed upon RNA translation within MP1-activated vRNP is identical to the products of translation of unbound RNA as follows from Fig. 4. The results obtained lead to assume that the structures of the protein coats of the “mixed” (heterologous) and homologous vRNPs are rather similar.

We demonstrated previously that protein–protein interactions between CP and MP1 play a key role upon the MP1-dependent activation of the RNA translation within viral particles or homologous vRNPs [15, 20]. The fundamental role in the interaction between CP and MP1 appears to belong to the C terminus fragment of PVX CP [21]. The results of the translational activation of heterologous RNAs within “mixed” artificial vRNPs serve as new evidence of the key role of the coat protein in this phenomenon.

Specific recognition of viral RNAs by the structural protein plays a key role in the encapsidation of viral RNA genomes during the assembly of a viral particle. Assembly signals interacting with coat proteins (origin of assembly, OAS) have been identified in the RNA molecules of a number of plant viruses (TMV, BMV, turnip crinkle virus) [22–25]. In particular, the significance of the 5’ terminus fragment of the genomic RNA in the processes of the assembly of the virus and replication of potexvirus RNA has been demonstrated [26].

Kwon *et al* . [27] were able to identify *in vitro* the origin of assembly of PVX within the 5’ terminus fragment of PVX RNA (51–84 nt) forming a stem-loop structure (SL1). Moreover, the regulatory elements that are required for the binding of RNA to the coat protein are shown to be located in the fragment 1–107 nt of PVX RNA [28]. The data obtained by an analysis of the translational properties of heterologous vRNPs allow one to assume that the initiation of *in vitro* assembly of heterologous vRNP also starts at the 5’ terminus RNA fragment and proceeds in the 5’–3’ direction. This conclusion is appropriate for potexvirus RNAs (NMV, PAMV). However, the signal for specific assemby of TMV is located in the 3’ terminus region; the tRNA-like 3’ terminal structure and elements of the polymerase gene play a role in assembly initiation in BMV. Mengo virus RNA (genus *Cardiovirus* , family *Picornaviridae* ) with a length of 8400 nt contains the virus-specific protein VPg [29] at its 5’ terminus; the protein is bound to the RNA via the phosphodiester bond; its 3’ terminus is polyadenylated [30]. It is not quite clear which sites are recognized by the PVX CP upon initiation of the “coating” of heterologous RNAs; although based on the results of translational activation, this process is likely to start at the 5’ terminus. It is most surprising that the translation of Mengo virus RNA, which has an internal translation origin site, is also inhibited upon binding to PVX CP and activated upon addition of MP1. Thus, it is plausible that the initiation of vRNP is determined by the protein at least in the case of PVX CP, and probably in the case of the papaya mosaic virus, too (according to Abouhaidar and Bancroft [31]). The assembly of the coat protein of PVX and heterologous RNAs is initiated at regions that differ considerably in terms of localization and structure from the ones in the case of RNA interaction with its “own” protein.

It can be assumed under the conditions of our experiment that PVX CP recognizes not a specific nucleotide sequence, but a certain structure of the 5’ terminus fragment of the RNA, which initiates the formation of vRNP.

## CONCLUSIONS

The incubation of various foreign heterologous RNAs with PVX CP *in vitro* has been shown to result in the formation of the vRNPs having morphology and translational properties similar to those of homologous vRNPs. It can be assumed that a protein coat will be formed upon interaction of a heterologous RNA and PVX CP similar to the coat of homologous particles in terms of its structure. The *in vitro* formation of heterologous vRNPs with the participation of PVX CP seems to be initiated at the 5’ terminus of an RNA molecule and to be independent of the specific nucleotide sequence of the 5’ terminus RNA fragment. As a result, PVX CP is capable of packaging foreign genetic material of different sizes into an artificial virus-like particle. Neither the heterologous nor homologous RNA within vRNP is accessible for ribosomes. However, it becomes translationally active upon incubation of the resulting vRNPs with PVX MP1. Binding of MP1 to one of the termini of the PVX virion induces conformation changes in the terminal subunits of the coat protein, which results in the destabilization (remodelling) and transition of the protein helix into a metastable state. The following MP1-dependent translational disassembly of PVX particles occurs rapidly and, most likely, in cooperation with the release of unbound RNA and protein subunits at early stages of the translation [20]. It is quite likely that the same mechanism is responsible for the translational activation of “mixed” vRNPs, with the participation of MP1, described above.

Coat proteins of plant viruses with a helical structure could be used to design and deliver into target organs artificial “hybrid” nanoparticles (vRNP) capable of *in vivo* disassembly under the control of various factors. 

## Abbreviations

PVX: Potato virus X
CP: coat protein
vRNP: viral ribonucleoprotein
MP: movement protein
BMV: Brome mosaic virus
PAMV: Potato aucuba mosaic virus
TMV: Tobacco mosaic virus
NMV: Narcissus mosaic virus
AltMV: Altenathera mosaic virus
